# Hydration Characteristics of Tricalcium Aluminate in the Presence of Nano-Silica

**DOI:** 10.3390/nano11010199

**Published:** 2021-01-14

**Authors:** Dapeng Zheng, Manuel Monasterio, Weipeng Feng, Waiching Tang, Hongzhi Cui, Zhijun Dong

**Affiliations:** 1 Key Laboratory for Resilient Infrastructures of Coastal Cities (Ministry of Education), Underground Polis Academy, College of Civil and Transportation Engineering, Shenzhen University, Shenzhen 518060, China; dpzheng2-C@my.cityu.edu.hk (D.Z.); microcanonico@gmail.com (M.M.); wp_feng@163.com (W.F.); dongzj@sziit.edu.cn (Z.D.); 2 Department of Architecture and Civil Engineering, City University of Hong Kong, Kowloon, Hong Kong 999077, China; 3 School of Architecture and Built Environment, The University of Newcastle, Callaghan, NSW 2308, Australia; patrick.tang@newcastle.edu.au

**Keywords:** nano-silica, tricalcium aluminate, pozzolanic reaction, C-A-S-H gel

## Abstract

Tricalcium aluminate (C_3_A) is the most reactive component of the Portland cement and its hydration has an important impact on the workability and early strength of concrete. Recently, nanomaterials such as nano-silica (nano-SiO_2_) have attracted much attention in cement-based materials because of its pozzolanic reactivity and the pore-filling effect. However, its influence on the hydration of C_3_A needs to be well understood. In this study, the hydration kinetics of C_3_A mixed with different percentages of nano-SiO_2_ were studied and compared with pure C_3_A. The hydration products were examined by different characterization techniques including XRD, XPS, and NMR spectroscopy and isothermal calorimetry analyses. The XRD results showed that the addition of nano-SiO_2_ promoted the conversion of the intermediate product C_4_AH_13_. The isothermal calorimetry results showed that the addition of nano-SiO_2_ significantly reduced the hydration exotherm rate of C_3_A from 0.34 to less than 0.1 mW/g. With the presence of nano-SiO_2_, the peaks for Q^1^ were observed in ^29^Si MAS-NMR measurements, and the content of Q^1^ increased from 6.74% to 30.6% when the nano-SiO_2_ content increased from 2 wt.% to 8 wt.%, whereas the proportion of Q^4^ gradually decreased from 89.1% to 63.6%. These results indicated a pozzolanic reaction provoked by the nano-SiO_2_ combined with aluminate structures generating C-A-S-H gel.

## 1. Introduction

It is well known that the macroscopic properties of a cement-based material are a consequence of how the constituent particles are arranged and held together at micro- and nano-scales. Many studies have been using nanomaterials such as nano-SiO_2_, nano-Al_2_O_3_, and nano-TiO_2_ to improve the microstructure performance of cement-based materials [1,2]. However, most of them focused the impact of nanomaterials on the hydration of tricalcium silicate (C_3_S) only [3], not on tricalcium aluminate (C_3_A), which is the most intense hydration mineral component in cement. Though the typical proportion of C_3_A in cement is only about 10 wt.% [4], it is the most reactive component of the Portland cement.

C_3_A, together with alite (C_3_S), belite (C_2_S), and ferrite (C_4_AF), are the main components of cement. Compared to C_3_S, the hydration of C_3_A is significantly faster, forming calcium hydroaluminates and other phases such as calcium hydroaluminate-ferrite, commonly called AFm [5]. However, the fast reaction, often named “flash setting” [6], will reduce the workability and strength of the final products, which is usually avoided by adding gypsum [4,5]. The sulfate in gypsum binds C_3_A, generating sulfoaluminates instead of calcium hydroaluminates [7]. The reactions of C_3_A with and without calcium sulfate are expressed as Equations (1)–(3) (in cement notation) [8,9]:

C_3_A + 3CS + 32H → C_6_AS_3_H_32_ (Ettringite) (1)

C_6_AS_3_H_32_ + 2C_3_A + 4H2O → 3C_4_ASH_12_ (Monosulphate) (2)

C_3_A + H → C_2_AH_8_ + C_4_AH_13_ → C_3_AH_6_ (3)

It is well-known that nano-SiO_2_ in cement can increase the density of the C-S-H gel and decrease the final porosity of the hydrated products [10,11]. Besides, it can reduce the amount of calcium hydroxide formation, as well as the setting time [12,13], and thus increase the hydration degree of cement [14,15] to obtain the best mechanical performance. All the above-mentioned advantages are achieved through the three mechanisms of nano-SiO_2_: The nucleation reaction, pozzolanic effect, and pore-filling effect. Nano-SiO_2_ can act as a nucleation site for C-S-H seeds, accelerating cement hydration. At the same time, nano-SiO_2_ particles can generate C-S-H gel by undertaking pozzolanic reaction that further intensifies the growth of C-S-H gels in the matrix and consequently increases the final density [16,17]. The pozzolanic reaction is the reaction between nano-SiO_2_ and calcium hydroxide, which allows the generation of C-S-H gel as expressed in Equations (4) and (5). In addition, the nano-SiO_2_ particles can fill up micro-pores to reduce the overall porosity of the final products [18,19].

SiO_2_ + H_2_O → Si(OH)_4_ (4)

Ca(OH)_2_ + Si(OH)_4_ → C-S-H (5)

However, to our knowledge, research about the effect of nano-SiO_2_ on C_3_A hydration remains limited. A few works in the literature postulate that the pozzolanic reaction of nano-SiO_2_ in a high-aluminum environment may generate C-A-S-H gel, similar to C-S-H gel, where some of the silicon tetrahedrons could be replaced by aluminum [20,21]. The main objective of this research is to study the effects of nano-SiO_2_ on the hydration of C_3_A and its hydrated products. It is worth noting that the Al/Si ratio used in this study was much higher than previous studies as the aluminum was not the substitute element [22].

In this research, X-ray diffraction (XRD) was used to determine the final hydration products of C_3_A with different contents of nano-SiO_2_. Besides, the isothermal calorimetry was chosen to examine the intensity and speed of different amounts of nano-SiO_2_ on the heat flow of C_3_A hydration. An X-ray photoelectron spectrometer (XPS) and nuclear magnetic resonance (NMR) were used to reveal the microstructure of the hydrated products. It is believed that the findings of this study can help to prove the existence of the C-A-S-H gel and other alterations in the hydration products of C_3_A with different contents of nano-SiO_2_.

## 2. Materials and Methods

### 2.1. Materials

**Figure 1:**
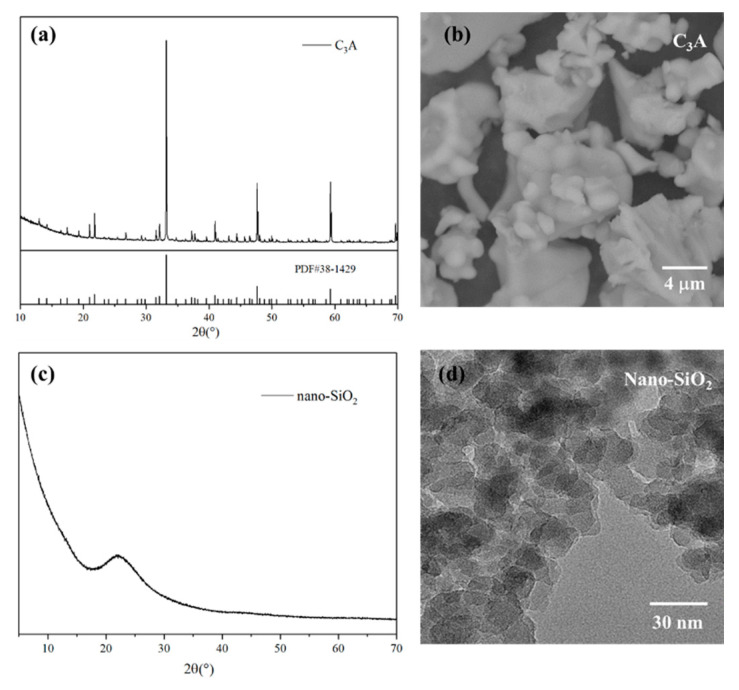
Mineral composition and particle characteristics of pure C_3_A and nano-SiO_2_ measured by (**a**) XRD diffraction pattern of pure C_3_A; (**b**) SEM photograph of C_3_A; (**c**) XRD diffraction pattern of nano-SiO_2_; (**d**) TEM photograph of nano-SiO_2_.

The C_3_A with a purity of 99 wt.%, employed in this experiment, was purchased from a company in Shanghai. The nano-SiO_2_ with a purity of 99.5 wt.% and an average particle size of 15 ± 5 nm was provided by Shanghai Macklin Biochemical Co., Ltd. Figure 1a shows the XRD pattern of the pure C_3_A with high and well-defined peaks, which proved to be cubic C_3_A according to PDF#38-1429. In addition, the SEM image in Figure 1b shows that the size of the pure C_3_A particle is about 10 μm. On the other hand, as seen in Figure 1c, the curve of pure nano-SiO_2_ shows a hump at 20–25°, indicating that the nano-SiO_2_ used in this study had noncrystallinity and high activity [23]. Therefore, XRD analysis can confirm that both nano-SiO_2_ and pure C_3_A were of high purity. Although the nano-SiO_2_ nanoparticles tended to agglomerate, an average size of about 10 nm could be measured from the TEM image, as shown in Figure 1d.

### 2.2. Sample Preparation

In this study, five mixes of pure C_3_A with different nano-SiO_2_ contents (0, 2, 4, 6, and 8 wt.%) were prepared as indicated in Table 1. The samples were prepared through the following procedures: Initially, the specific amounts of C_3_A and nano-SiO_2_ in powder form were weighed and premixed. Then, the mixture was placed in a glass flask filled with deionized water and mixed continuously by a magnetic stirrer for 72 h. The speed of stirring was 800 rpm and the liquid-to-solid ratio (L/S) was kept constant at 50. It is worth emphasizing that the glass flask was always filled with nitrogen during the 72 h hydration process to prevent possible carbonization and impurities in the air. After 72 h, the samples were removed from the stirring platform and filtered using a 7 cm filter paper and a suction filter glass. Then, the samples were placed into a vacuum oven and allowed to dry for 7 days at a temperature of 40 °C. The dried samples were kept sealed for later testing.

### 2.3. Methods

X-ray diffraction (XRD, D8 Advance, Bruker, Germany) was used to determine the mineralogical composition of raw materials and hydration products of C_3_A (with and without nano-SiO_2_). A scanning rate of 0.08 °/s from 5° to 70° with Cu Kα radiation (λ = 1.5418 Å) was used, as well as a screen to prevent high background at small degrees.

Transmission Electron Microscopy (TEM, TALOS F200X, Thermo Fisher Scientific, Waltham, Massachusetts, USA) and Scanning Electron Microscopy (SEM, TM 250 FEG, Thermo Fisher Scientific, Waltham, Massachusetts, USA) were employed to determine the particle characteristics of the raw samples.

A Thermal Activity Monitor (TAM-AIR, TA Instruments, New Castle, Delaware, USA), equipment for isothermal calorimetry, was employed to analyze the heat flow produced during the hydration of the samples. The samples were prepared using 0.5 g of C_3_A and the corresponding percentages of nano-SiO_2_ with a water-to-solid ratio of 5. Prior to the hydration process, the samples in powder form were kept inside the device for 3–4 h until the calorimeter was stabilized. Then, the distilled water was added and the mixtures were stirred for 30 s to begin the hydration.

The solid-state Nuclear Magnetic Resonance (MAS-NMR, JEOL-600, Japan) technique was used in two different modes. The first mode was ^29^Si MAS-NMR that employed single-pulse decoupling including 1024 scans with a relaxation delay of 60 s. The probe was 8 mm in diameter and the spinning speed was 5000 rpm. The second mode was ^27^Al MAS-NMR, a single pulse with 1000 scans, and 10 s of relaxation time. The probe was 3.2 mm in diameter and the spinning speed was 12,000 rpm. The references used to calibrate the peaks were TSPA and AlK (SO_4_) for ^29^Si and ^27^Al, respectively.

The X-ray photoelectron spectrometer (XPS, Thermo escalab XI+) was employed to verify the molecular structure of the hydration products and record the measurements with a monochromatic Al Kα (hν = 1486.6 eV) X-ray source, employing a flare area of 650 µm, calibrated by 284.8 eV C1s. A constant analyzer pass energy of 20 eV was applied.

## 3. Results and Discussion

### 3.1. Mineral Composition Analysis

**Figure 2:**
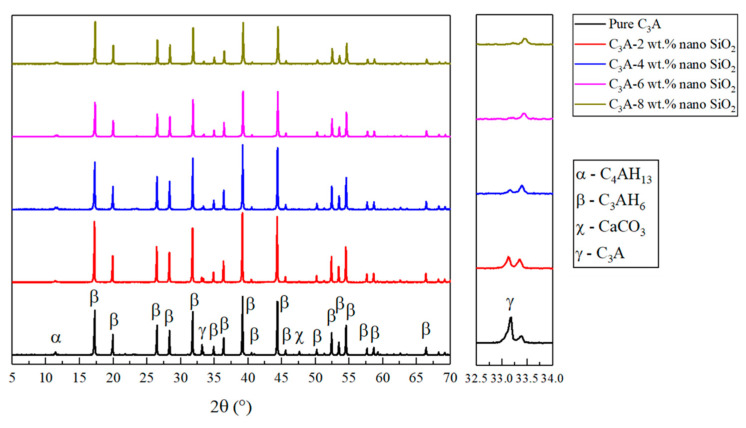
XRD analysis of all the samples to observe the evolution of peaks related to the hydration of the samples.

The XRD analysis of the hydrated samples can be seen in Figure 2. In agreement with the literature [24,25], the majority of the peaks found in the results were related to Katoite (written as Ca_3_Al_2_(OH)_12_ or C_3_AH_6_), which is one of the final forms of the C_3_A hydration, indicating a good reaction process of C_3_A. Another peak at 11.5° found in all the samples was related to C_4_AH_13_, which is an intermediate form during the hydration of C_3_A. The small peak at 47.5° was only found in the hydration products of pure C_3_A, which may be related to the carbonation of C_3_A hydration products. Besides, another peak at 33.2° is associated with anhydrate C_3_A, indicating that the hydration of pure C_3_A was incomplete. The intensity of this peak was reduced after adding 2 wt.% of nano-SiO_2_ and even smaller when the nano-SiO_2_ content increased to 4 wt.%. Furthermore, this peak disappeared completely in the samples of C_3_A with 6 wt.% and 8 wt.% nano-SiO_2_. This result substantiates the promotion effect of nano-SiO_2_ on the hydration process of C_3_A.

### 3.2. Hydration Exothermic Analysis

**Figure 3:**
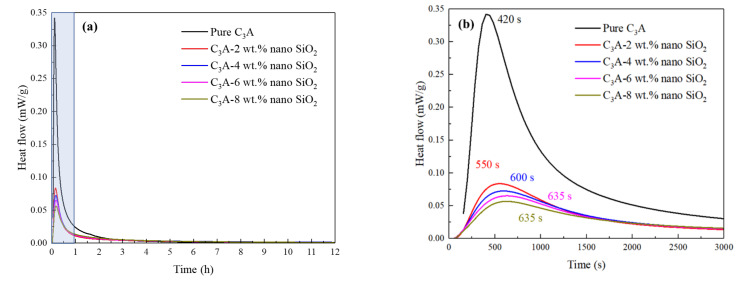
Heat flow rate of C_3_A samples with different nano-SiO_2_ contents in (**a**) 12 h (the blue area is enlarged and displayed as (**b**)) and (**b**) 3000 s (50 min). The number in (**b**) is the time needed to reach the maximum heat flow rate, in seconds.

The effect of nano-SiO_2_ on the hydration heat release rate of C_3_A was studied using the isothermal calorimetry, and the results can be seen in Figure 3. De Jong et al. [26] previously found a second exothermic peak observed in the C_3_A hydration process after adding amorphous silica, which appeared in the first hours of hydration but being at later stage when the amorphous silica content increased. However, as shown in Figure 3a, this study showed different results. It can be seen from Figure 3b that the pure C_3_A sample showed the highest reactivity and the peak in hydration heat release rate occurred at 420 s. When 2 wt.% nano-SiO_2_ was added to the C_3_A hydration system, the hydration heat release rate of C_3_A was greatly reduced from 0.34 to less than 0.1 mW/g. The hydration rate can be further reduced with increasing nano-SiO_2_ content, but the significance is not obvious. With 2 wt.% nano-SiO_2_, the hydration exothermic peak was delayed by more than 2 min. In addition, a further delay was noted with the increase in the nano-SiO_2_ content. Xu et al. [3] stated that the addition of nano-SiO_2_ will accelerate the rate of heat release in the early stage of C_3_S hydration. However, the opposite phenomenon occurred during the C_3_A hydration process. The reduction in heat release can be attributed to two reasons: (1) The hydration reaction rate of C_3_A was much higher than that of C_3_S, and the surface of the C_3_A particles was adsorbed with a large amount of nano-SiO_2_ with high specific surface area, which would reduce the contact area between C_3_A and water, thus slowing down the reaction rate [27]; (2) the surface of C_3_A particles was covered by the C-A-S-H gels (generated by the pozzolanic reaction of nano-SiO_2_ combined with C_3_A), thereby reducing the reaction rate. This is consistent with the phenomenon that the appearance time of the C_3_A hydration exothermic peak continuously delayed as the amount of nano-SiO_2_ increased. Hou et al. [28] explored the influence of nano-SiO_2_ on the hydration process of C_3_A-gypsum and C_3_A-C_3_S-gypsum systems, and obtained similar conclusions. They believed that the nano-SiO_2_ adsorbed on the surface of C_3_A due to the electrostatic effect is the reason for the delayed hydration. At the same time, the C-S-H gel generated by the pozzolanic effect of nano-SiO_2_ can cover the surface of C_3_A, which will also inhibit the hydration heat release rate of C_3_A.

### 3.3. X-ray Photoelectron Spectroscopy Results

**Figure 4:**
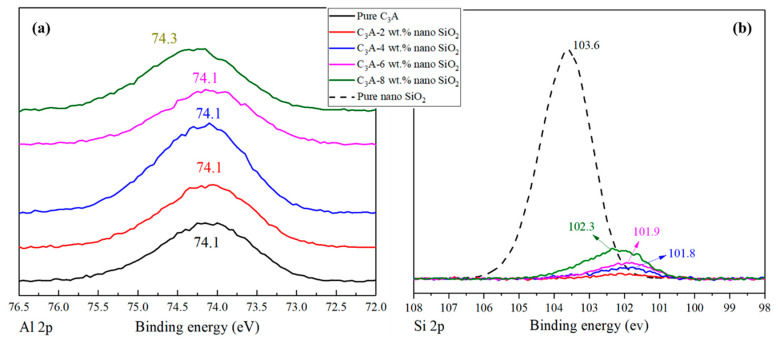
XPS results of (**a**) Al 2p and (**b**) Si 2p for hydration products of pure C_3_A and C_3_A with different nano SiO_2_ contents.

The XPS was performed to analyze the binding energies of Al 2p and Si 2p in the hydration products of C_3_A with different nano-SiO_2_ contents. For Al 2p, previous work has indicated that the binding energy of octahedral coordinated aluminum is generally higher than that of the tetrahedral form [29]. It can be seen from Figure 4a that the Al 2p binding energy of the pure C_3_A hydration products was around 74.1 eV, which can be related to C_3_AH_6_ [30,31]. All the samples containing nano SiO_2_, except C_3_A-8 wt.%, gave similar results showing a unique peak around 74.1 eV. The sample of C_3_A with 8 wt.% nano-SiO_2_ presented a peak around 74.3 eV. It can be inferred that the amount of 8 wt.% nano-SiO_2_ was enough to influence the binding energy of C_3_A hydration products. It also indicated that the reaction between nano-SiO_2_ and C_3_A could create an Al-O-Si bond as Al would migrate to Si due to its high electronegativity, thus increasing the Al 2p binding energy [32]. On the other hand, the binding energy of Si 2p in all hydration products is shown in Figure 4b. It can be seen from the figure that the Si 2p binding energy of pure nano-SiO_2_ is 103.6 eV, while the Si 2p binding energy of C_3_A with 2 wt.% nano-SiO_2_, however, showed a very low intensity, almost being a plateau. This phenomenon indicates that there is Al element insertion in the silicon chain, which leads to a substantial decrease in Si 2p binding energy. Besides, the Si 2p binding energy rebounded with the amount of nano-SiO_2_ increased. When the nano-SiO_2_ content increased to 4 wt.%, 6 wt.%, and 8 wt.%, the Si 2p binding energy rebounded to 101.8, 101.9, and 102.3 eV, respectively, and the peaks became more obvious. This phenomenon could prove the formation of Al-O-Si bonds when nano-SiO_2_ was added to C_3_A. Overall, the Si 2p results are consistent with the Al 2p binding energy results, and are in good agreement with the previous findings [33,34].

### 3.4. Structural Changes Observed by Nuclear Magnetic Resonance

**Figure 5:**
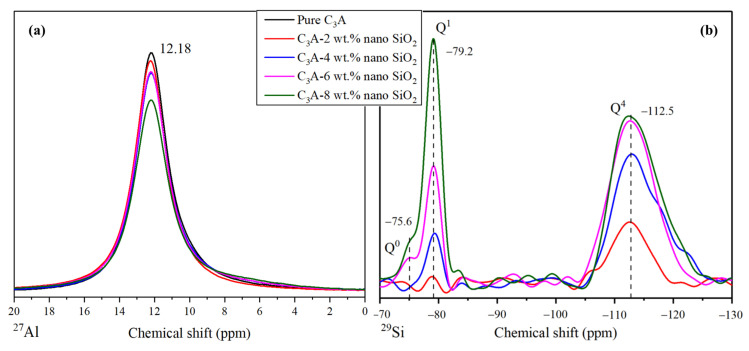
MAS-NMR signal obtained for (**a**) ^27^Al and (**b**) ^29^Si. ^29^Si measurement shows a large number of peaks; meanwhile, ^27^Al shows one peak uniquely.

The NMR spectroscopy was performed with the intention of finding relevant information to prove a possible alteration in the hydration products structures brought by nano-SiO_2_ [35]. Figure 5 shows the ^29^Si and ^27^Al MAS-NMR results of all samples, in order to reveal the effect of nano-SiO_2_ on the structure of C_3_A hydration products. As shown in Figure 5a, all the hydration samples showed similar ^27^Al MAS-NMR results with a unique peak at 12.18 ppm, which is related to the octahedral aluminum configuration in the C_3_AH_6_ component [8,36,37]. The absence of any shift in the peak can lead to the conclusion that the presence of nano-SiO_2_ would not alter the original structure formed by the C_3_A hydration [35].

**Figure 6:**
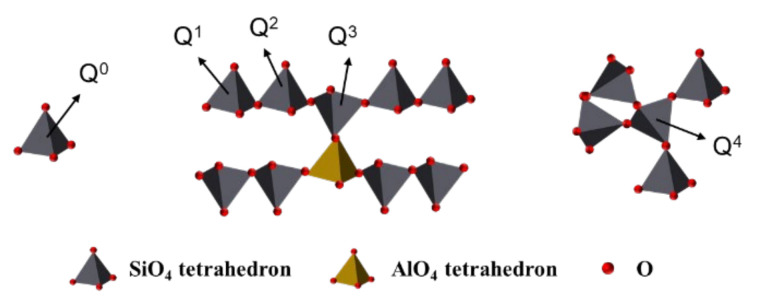
Silicon chain structure in C-S-H gel.

However, the ^29^Si MAS-NMR results shown in Figure 5b are different from the ^27^Al MAS-NMR results. The peaks were labeled as Q^n^, where “n” is the number of similar tetrahedrons connected in the molecule. For example, Q^0^ refers to a silicon tetrahedral configuration completely insolated to other silicon, whereas Q^2^ refers to a silicon tetrahedral configuration connected with another two silicon tetrahedrons, forming a chain of silicon, as illustrated in Figure 6. According to the ^29^Si MAS-NMR results, the two peaks observed around −79.2 and −112.5 ppm were Q^1^ and Q^4^, respectively. In this context, Q^1^ implies the existence of a silicon tetrahedron in the final position of a chain [38,39,40,41]. Sometimes, this peak is found in a more negative value (around −81 ppm) [39,42]. This chemical shift is in agreement with the presence of aluminum in the environment of silicon tetrahedra to reach more positive ppm values [42,43]. This is a good indicator of the generation of dimers, combining silicon and aluminum tetrahedra. The intensity of this peak grew considerably with nano-SiO_2_ content, owing to the larger amount of dimers generated within the structure. The peak Q^4^ refers to the presence of a “three-dimensional” net of silicon tetrahedra in the sample [44,45], and is strongly related to nano-SiO_2_ [23,46]. In the Q^1^ peak, there is a shift to more positive values due to the presence of aluminum within the close environment of the silicon tetrahedron. Additionally, new peaks were found in some of the samples. The Q^0^ peak appeared in hydration products of C_3_A with 6 wt.% and 8 wt.% nano SiO_2_, which is associated with silicon tetrahedra completely insolated. Due to this peak only being found in samples with a high percentage of nano-SiO_2_, this could imply that some silicon ions from the dissolution of the nano-SiO_2_ were not integrated into the C-A-S-H structure.

**Figure 7:**
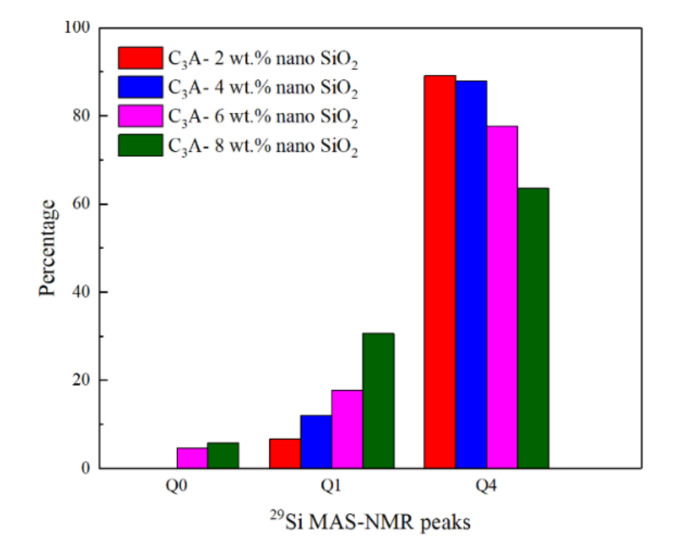
Percentages of different Q^n^ peaks, calculated by Gaussian deconvolution from the ^29^Si MAS-NMR measurements.

The percentages of different Q^n^ peaks obtained by Gaussian deconvolution are shown in Figure 7. It is quite clear to show that the existence of Q^0^ depends on nano-SiO_2_ content. On the other hand, the proportion of Q^1^ increased rapidly from 6.74% to 30.6% when the amount of nano-SiO_2_ increased from 2 wt.% to 8 wt.%. However, the proportion of Q^4^ gradually decreased from 89.1% to 63.6% correspondingly. This finding implies that the higher the nano-SiO_2_ content, the more silicon dimers in hydration products can be formed. Additionally, the positions of the Q^4^ peaks were slightly shifted to more negative values from −112.63 to −113.53 ppm as the nano-SiO_2_ content increased from 2 to 8 wt.%. This observation is in agreement with the trend of the position of the pure nano-SiO_2_ Q^4^ peak, around −115 ppm [23]. In addition, not all the silicon tetrahedra would be influenced by aluminum with a high content of nano-SiO_2_, and the shift to more positive values created by the aluminum would become weaker.

Owing to the C-A-S-H gel being amorphous, its presence can only be revealed by the apparition of the Q^1 29^Si MAS NMR peak and the shifted Q^4^ to more positive values under the influence of aluminum. Based on the findings from this study, it is safe to say that nano-SiO_2_ can promote the apparition of C-A-S-H gel in the final hydration products of C_3_A, besides the C_3_AH_6_ structure.

## 4. Conclusions and Recommendations

This study examined the effect of nano-SiO_2_ on the hydration of C_3_A in cement. According to the test results, the following conclusions are drawn:

(1) The addition of nano-SiO_2_ can promote the hydration degree of C_3_A while significantly reducing the heat release rate of C_3_A hydration from 0.34 to less than 0.1 mW/g, and the occurrence time of the hydration exothermic peak was delayed by more than 2 min. The main reasons are probably the surface of C_3_A being adsorbed by nano-SiO_2_ and/or covered by the C-A-S-H gel (formed by the pozzolanic hydration reaction of nano-SiO_2_ and C_3_A) at an early age, thereby reducing the contact area of C_3_A with water.
(2) The reaction between nano-SiO_2_ and C_3_A can establish Si-O-Al bonds and generate C-A-S-H gels. The chemical shifts in Al 2p and Si 2p both confirm this conclusion. In addition, ^29^Si MAS-NMR results showed that Q^1^ appeared after the nano-SiO_2_ was added to the C_3_A hydration system. With the nano-SiO_2_ content increased, the proportion of Q^1^ in the hydration product increased from 6.74% to 30.6%, while the proportion of Q^4^ gradually decreased from 89.1% to 63.6%.
(3) The addition of nano-silica can promote the hydration reaction rate of C_3_S while delaying the hydration of C_3_A. For the two hydration systems of C_3_A and C_3_S, the addition of nano-SiO_2_ has shown completely different effects, and the influence mechanism of nano-SiO_2_ in the two different hydration processes needs further exploration.

## Figures and Tables

**Table 1:** Proportion of different components used during the preparation of the samples.

Sample Name	C_3_A (g)	Nano-SiO_2_ (g)	Water (g)	L/S
Pure C_3_A	1	0	50	50
C_3_A-2 wt.% nano SiO_2_	1	0.02	51	50
C_3_A-4 wt.% nano SiO_2_	1	0.04	52	50
C_3_A-6 wt.% nano SiO_2_	1	0.06	53	50
C_3_A-8 wt.% nano SiO_2_	1	0.08	54	50

## Data Availability

Data is contained within the article.

## Abbreviations

C_3_A	Tricalcium aluminate
C_3_S	Tricalcium silicate, alite
C_2_S	Dicalcium silicate, belite
C_4_AF	Tetracalcium aluminate, ferrite
CS	Calcium sulfate, CaSO_4_
C-S-H	Calcium silicate hydrate
C-A-S-H	Calcium silicoaluminate hydrate
C_6_AS_3_H_32_, Aft	Ettringite
3C_4_ASH_12_, AFm	Monosulfate
Ca_3_Al_2_(OH)_12_, or C_3_AH_6_	Calcium aluminium hydrate
C_4_AH_13_/C_2_AH_8_	Calcium aluminium hydrate
